# Deregulation of the *Os*miR160 Target Gene *OsARF18* Causes Growth and Developmental Defects with an Alteration of Auxin Signaling in Rice

**DOI:** 10.1038/srep29938

**Published:** 2016-07-21

**Authors:** Jian Huang, Zhiyong Li, Dazhong Zhao

**Affiliations:** 1 Department of Biological Sciences, University of Wisconsin-Milwaukee, Milwaukee, WI 53211, USA

## Abstract

MicroRNAs (miRNAs) control gene expression as key negative regulators at the post-transcriptional level. MiR160 plays a pivotal role in *Arabidopsis* growth and development through repressing expression of its target *AUXIN RESPONSE FACTOR* (*ARF*) genes; however, the function of miR160 in monocots remains elusive. In this study, we found that the mature rice miR160 (*Os*miR160) was mainly derived from *OsMIR160a* and *OsMIR160b* genes. Among four potential *Os*miR160 target *OsARF* genes, the *OsARF18* transcript was cleaved at the *Os*miR160 target site. Rice transgenic plants (named *mOsARF18*) expressing an *Os*miR160-resistant version of *OsARF18* exhibited pleiotropic defects in growth and development, including dwarf stature, rolled leaves, and small seeds. *mOsARF18* leaves were abnormal in bulliform cell differentiation and epidermal cell division. Starch accumulation in *mOsARF18* seeds was also reduced. Moreover, auxin induced expression of *OsMIR160a*, *OsMIR160b*, and *OsARF18*, whereas expression of *OsMIR160a* and *OsMIR160b* as well as genes involved in auxin signaling was altered in *mOsARF18* plants. Our results show that negative regulation of *OsARF18* expression by *Os*miR160 is critical for rice growth and development via affecting auxin signaling, which will advance future studies on the molecular mechanism by which miR160 fine-tunes auxin signaling in plants.

MicroRNAs (miRNAs), which are small (~21 nucleotides) non-coding RNAs, act as critical negative regulators by binding to mRNA complementary sequences for mRNA destabilization and translational inhibition in both plants and animals^1^,^2^,^3^,^4^,^5^. In plants, primary miRNAs (pri-miRNA) are transcribed from *MIRNA* (*MIR*) genes. Stem-loop segments derived from pri-miRNAs are cleaved by RNase III-type endonucleases (also known as Dicers) to produce paired precursor miRNAs (pre-miRNA). After liberation of miRNA duplexes, mature miRNAs approximately 21-nucleotide long direct RNA-induced silencing complexes (RISC) to bind to their target mRNAs by complementary match, leading to cleavage or translational inhibition. In the model species *Arabidopsis*, the general roles of miRNAs are recognized by analysis of mutants that are impaired in miRNA biogenesis, while the functions of specific miRNAs have been established by either expressing miRNA-resistant versions of target genes or overexpressing miRNAs. The roles of miRNAs are also investigated by loss-of-function analyses of *MIR* genes or by interfering with mature miRNAs^6^,^7^,^8^. Despite identification of a large number of miRNAs from a variety of plant species, the functions of most individual miRNAs remain unclear, particularly in plant species other than *Arabidopsis*.

In rice (*Oryza sativa* L.), miRNAs play a remarkably wide number of roles in growth and development. Rice has six *DICER*-*LIKE* (*OsDCL*) genes with *Os*DCL1 being the major enzyme for producing mature miRNAs^9^,^10^,^11^. Strong *OsDCL1* loss-of-function mutants are not viable, while weak lines exhibit pleiotropic defects. The rice *AGO* gene *MEIOSIS ARRESTED AT LEPTOTENE1* (*MEL1*) is required for meiosis^12^. Loss-of-function studies of WAF1, an ortholog of *Arabidopsis* HEN1, have shown that WAF1 is responsible for shoot development via maintaining miRNAs and trans-acting-small interfering RNAs (ta-siRNAs)^13^. Characterization of the *IPA1* (*Ideal Plant Architecture 1*) trait locus has revealed that the *Os*miR156-controlled *OsSPL14* is critical for grain yield^14^. In addition, *Os*miR156 may target *OsSPL16* to control grain size, shape, and quality^15^. Furthermore, the gradually increased expression of *Os*miR156 in leaves is important for leaf development^16^. Overexpression of *Os*miR172 represses expression of *AP2*-like genes, which consequently causes abnormal floral meristem identity and defects in flower organ and seed development^17^,^18^. *OsTIR1* and *OsAFB2* are predicted targets of *Os*miR393. Transgenic plants overexpressing *Os*miR393a/b have defects similar to those observed in auxin signaling mutants^19^. Although various miRNAs have been found in rice^20^,^21^, it is imperative to study functions of individual *Os*miRNAs in rice growth and development, particularly those of agricultural importance.

MiR160 is essential for plant growth and development^6^. In *Arabidopsis*, miR160 targets *AUXIN RESPONSE FACTOR 10* (*ARF10*), *ARF16*, and *ARF17*. Expression of the miR160-resistant version of *ARF16* (*mARF16*) results in reduced fertility and less lateral roots^22^. Plants expressing *mARF17* exhibited pleiotropic defects in vegetative and reproductive development^23^. Analyses using similar approaches have revealed that the miR160-controlled *ARF10* is essential for seed germination and many post-embryonic growth and developmental processes through the regulation of both auxin and ABA signaling^24^. Recent studies have shown the dormancy of *mARF10* and *mARF16* seeds is increased^25^. In tomato, *Sly*miR160 and the *Sly*miR160a target *SlyARF10* are required for floral organ and early fruit development^26^,^27^. Moreover, in soybean, miR160 is involved in auxin and cytokinin signaling during nodulation^28^. Although miR160 is conserved in plants^29^,^30^, the role of miR160 in monocots is unknown. In this report, we examined expression of six *OsMIR160* genes and found that the mature *Os*miR160 was mainly derived from *OsMIR160a* and *OsMIR160b* genes. In addition, the *OsARF18* transcript was cleaved at the *Os*miR160 target site. We generated transgenic rice plants (named *mOsARF18*) that expressed an *Os*miR160-resistant version of *OsARF18*. Phenotypic analyses revealed that *mOsARF18* plants showed pleiotropic defects in growth and development. Furthermore, auxin treatment induced expression of *OsMIR160a*, *OsMIR160b*, and *OsARF18*, whereas expression of *OsMIR160a* and *OsMIR160b* as well as other genes involved in auxin signaling was altered in *mOsARF18* plants. Our results support the idea that deregulation of the *Os*miR160 target gene *OsARF18* leads to abnormal growth and development in rice through affecting the auxin signaling.

## Results

### Expression of *OsMIR160* genes and *Os*miR160 target *OsARF* genes in rice

**Figure 1:**
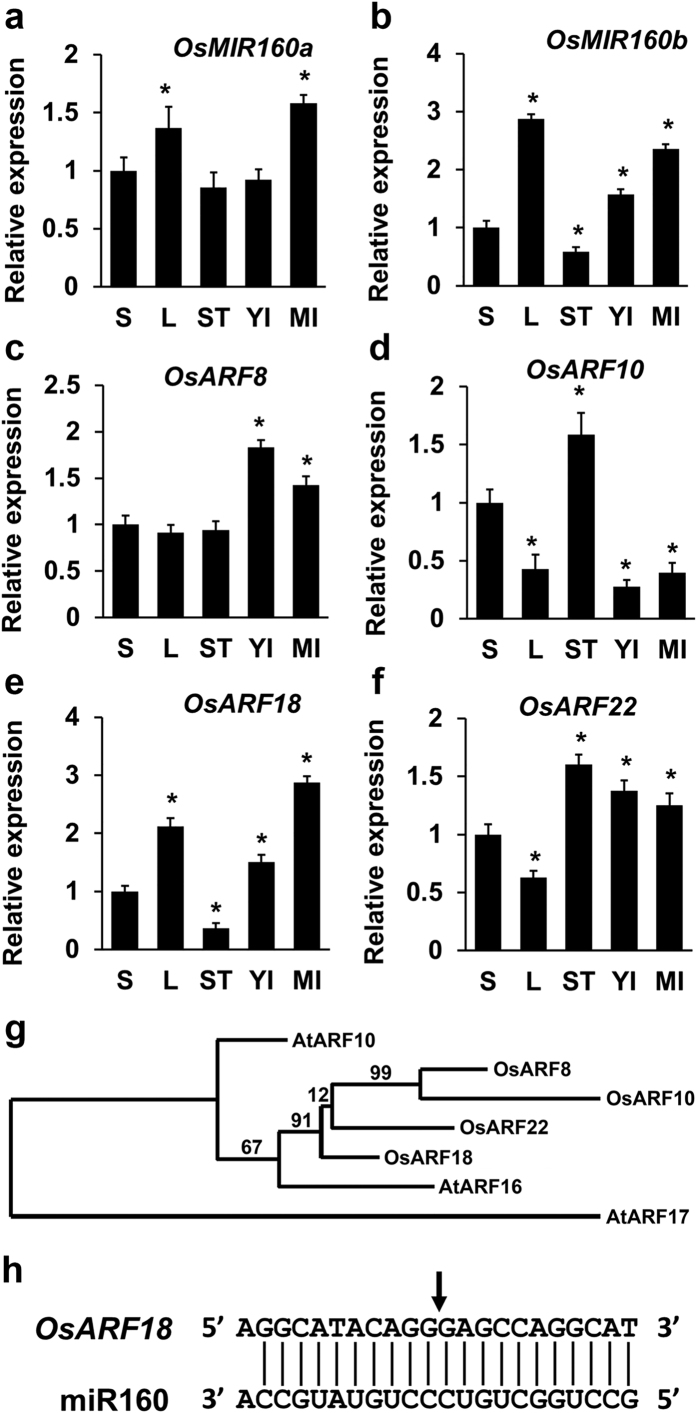
Expression analysis of *OsMIR160a*, *OsMIR160b*, and *Os*miR160 potential target *OsARFs*. (**a,b**) Quantitative real time RT-PCR (qRT-PCR) results showing expression of *OsMIR160a* (**a**) and *OsMIR160b* (**b**). (**c–f**) qRT-PCR) results showing expression of *Os*miR160 potential target genes: *OsARF8* (**c**), *OsARF10* (**d**), *OsARF18* (**e**), and *OsARF22* (**f**). S: seedling (7-day old), L: leaf, ST: stem, YI: young inflorescence, and MI: mature inflorescence. Gene expression levels in other organs were normalized based on expression observed in seedling. Stars indicate significant difference (P < 0.01). (**g**) An unrooted phylogenetic tree constructed by the Maximum-Likelihood method showing miR160 target ARFs in *Arabidopsis* and rice. (**h**) The 5′ RACE result showing that *OsARF18* is a target of *Os*miR160. The arrow points to the *Os*miR160-directed cleavage site at the *OsARF18* transcript.

Rice has six *OsMIR160* (*OsMIR160a* to *OsMIR160f*) genes. Four of six mature *Os*miR160s (*Os*miR160a to *Os*miR160d) have identical sequences, while the other two (*Os*miR160e and *Os*miR160f) each differs by a single nucleotide (Fig. S1a). Our RT-PCR results showed that expression of *OsMIR160a* and *OsMIR160b* genes was significantly higher than that of the other four *OsMIR160* genes in leaf (L), young inflorescence (YI), mature inflorescence (MI), and stem (ST) (Fig. S1b). Moreover, quantitative real-time RT-PCR (qRT-PCR) revealed that *OsMIR160a* and *OsMIR160b* genes had higher expression levels in leaf and inflorescences than that in seedling and stem (Fig. 1a,b). Our studies suggest that the mature *Os*miR160 might be mainly derived from *OsMIR160a* and *OsMIR160b* genes.

Our analyses using the psRNATarget web server^31^ showed that *OsARF8* (*Os*02g41800), *OsARF10* (Os04g43910), *OsARF18* (*Os*06g47150), and *OsARF22* (*Os*10g33940) were potential *Os*miR160 target genes. Further phylogenetic analysis found that all four targets were similar to *Arabidopsis* ARF10 and ARF16, whereas *Os*ARF18 had the highest similarity to the *Arabidopsis* ARF16 (Fig. 1g; Fig. S2). Our qRT-PCR results demonstrated that the expression of *OsARF8* mainly occurred in young and mature inflorescences (Fig. 1c), while *OsARF10* was primarily expressed in seedling and stem (Fig. 1d). *OsARF18* was predominantly expressed in leaf as well as young and mature inflorescences (Fig. 1e). Expression levels of *OsARF22* appeared similar in all examined organs except in leaf (Fig. 1f). A previous study using the “degradome sequencing” approach has shown that cleavage frequencies associated with *OsARF18* and *OsARF22* are the highest, but frequencies for *OsARF8* and *OsARF10* are extremely low^32^. We further identified that the *OsARF18* transcript was cleaved at the *Os*miR160 target site using a gene-specific 5′ RACE (Fig. 1h). Therefore, our results suggest that *OsARF18* (*Os*06g47150) is a promising target gene of *Os*miR160.

### Negative regulation of *OsARF18* by *Os*miR160 is essential for rice growth and development

**Figure 2:**
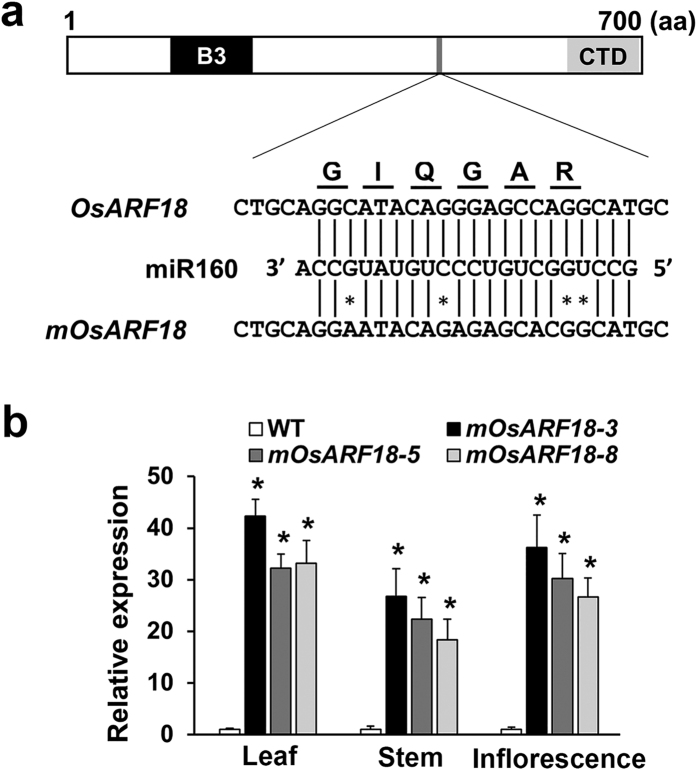
Generation of transgenic rice plants expressing an *Os*miR160-resistant version of *OsARF18* (*mOsARF18*). (**a**) Domain structure of the *Os*ARF18 protein. The B3 DNA binding domain (B3) and the C-terminal dimerization domain (CTD) are indicated in black and gray, respectively. The *Os*miR160 complementary (or target) sequence in the *OsARF18* mRNA and the corresponding region of amino acid sequence (GIQGAR) are shown. The silent mutations were created in *mOsARF18* by introducing silent mutations (indicated by*). (**b**) qRT-PCR results showing that expression levels of un-cleaved *OsARF18* were significantly (*P < 0.01) increased in leaf, stem, and inflorescence of three examined independent *mOsARF18* transgenic lines.

To investigate the function of *Os*miR160 in rice, we chose its promising target *OsARF18* to generate transgenic plants expressing an *Os*miR160-resistant version of *OsARF18* (resulting plants named *mOsARF18*) in the *Oryza sativa* Japonica cv. Nipponbare background (Fig. 2a). Our qRT-PCR results show that the expression of un-cleaved *OsARF18* was significantly increased in leaf, stem, and inflorescence of three examined *mOsARF18* transgenic lines (Fig. 2b).

**Figure 3:**
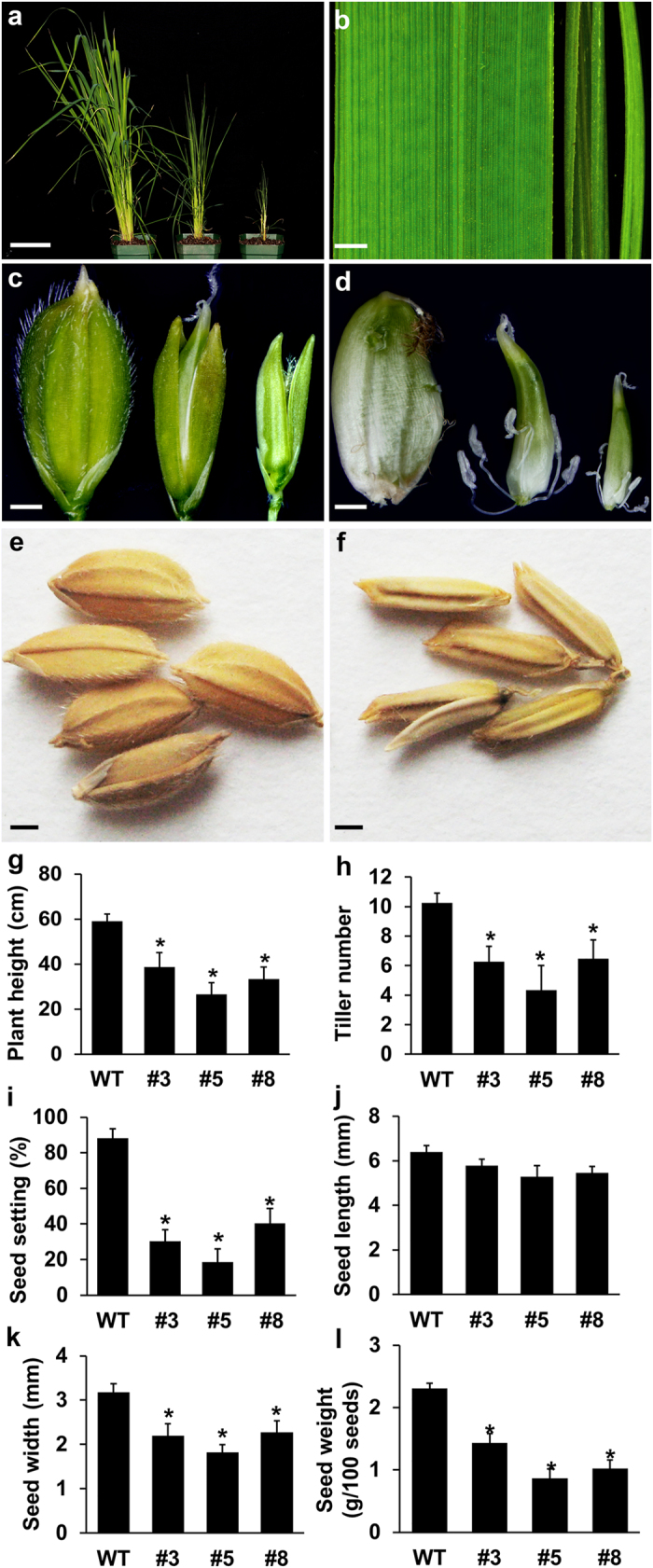
*mOsARF18* transgenic plants exhibited pleiotropic defects in growth and development. (**a**) Forty-day old wild-type (left), *mOsARF18-3* (middle), and *mOsARF18-5* (right) plants. Bar: 10 cm. (**b**) The fifth leaves of wild-type (left), *mOsARF18–3* (middle), and *mOsARF18-5* (right) plants. Bar: 1 mm. (**c,d**) Ten-DAP (Days After Pollination) wild-type (left), *mOsARF18-3* (middle), and *mOsARF18-5* (right) developing seeds. Bars: (**c,d**) 1 mm. Lemma and palea were removed in (**d**) to show attached stamens. (**e,f**) Wild-type (**e**) and *mOsARF18-3* (**f**) mature seeds. Bars: (**e,f**) 1 mm. (**g,h**) Sixty-day old transgenic plants showed significantly (*P < 0.01) decreased pant height (**g**) and tiller numbers (**h**) than that of wild type plants. (**i–l**) Mature transgenic plant seeds showed no difference in seed length (**j**), but significantly (*P < 0.01) decreased seed setting (**i**), width (**k**) and weight (**l**).

**Figure 4:**
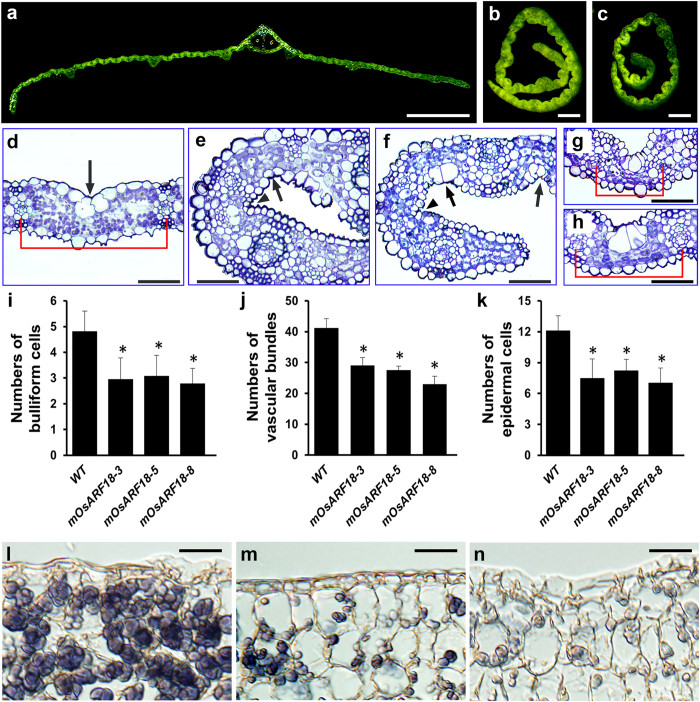
*mOsARF18* plants were abnormal in leaf cell division and differentiation as well as starch accumulation in seeds. (**a–c**) Hand cutting of the fifth leaves of wild-type (**a**), *mOsARF18-3* (**b**), and *mOsARF18-5* (**c**) plants. Bars: (**a**) 500 μm; (**b,c**) 200 μm. (**d–h**) Semi-thin sections of the fifth leaves of wild-type, *mOsARF18-3*, and *mOsARF18-5* plants: (**d**) wild type; (**e,g**) *mOsARF18-3*; and (**f,h**) *mOsARF18-5*. Arrows indicate bulliform cells. Arrowheads indicate the lack of typical bulliform cells. Red brackets indicate regions between two vascular bundles for epidermal cell counting. Bars: (**d–h**) 50 μm. (**i–k**) Numbers of bulliform cells (**i**), total vascular bundles (**j**), and abaxial epidermal cells between two vascular bundles (**k**) were significantly (*P < 0.01) decreased in leaves of *mOsARF18-3*, *mOsARF18-5*, and *mOsARF18-8* transgenic lines in comparison with wild-type plants. (**l**) Endosperm in 10-DAP (Days After Pollination) wild-type developing seeds showing strongly stained starch granules by I-KI (iodine-potassium iodide). (**m,n**) Endosperm in 10-DAP *mOsARF18-3* (**b**) and *mOsARF18-5* (**c**) developing seeds showing small and weakly stained starch granules. Bars: (**l–n**) 20 μm.

Eleven out of 19 *mOsARF18* independent transgenic lines exhibited strong but similar defects in both growth and development, while the rest of lines showed mild phenotypes. Compared to wild-type plants, *mOsARF18* lines were dwarf and formed less tillers (Fig. 3a,g,h). *mOsARF18* plants produced short and rolled leaves (Fig. 3b; Fig. S3; Fig. 4a–c). *mOsARF18* plants were also defective in reproduction, as indicated by abnormal flower and seed development (Fig. 3c–f). The lemma and palea did not enclose flowers (Fig. 3c). After fertilization, stamens remained attached to developing seeds, suggesting abnormal senescence of stamens (Fig. 3d). Moreover, *mOsARF18* lines showed reduced seed setting (Fig. 3i). Although no change in seed length (Fig. 3e,f,j), *mOsARF18* seeds had reduced width and weight when compared with wild type (Fig. 3e,f,k,l). Our results suggest that deregulation of *OsARF18* results in abnormal growth and development in rice.

### Cell division and differentiation were abnormal in *mOsARF18* leaves

To further examine leaf defects in *mOsARF18* plants, we analyzed the structure of the fifth mature leaf via cross-section. Compared with wild type, *mOsARF18* lines produced rolled leaves (Fig. 4a–c). Bulliform cells are specialized epidermal cells between two vascular bundles on the adaxial blade. Bulliform cells are large and highly vacuolated, which are important for leaf rolling through turgor pressure regulation^33^. Wild-type bulliform cells were arranged in groups of approximately 5 cells, among which the middle bulliform cell was larger than others along both sides (Fig. 4d,i). However, in *mOsARF18* leaves, we observed reduced number and size of bulliform cells, or bulliform cells were absent (Fig. 4e,f). Further statistical analysis showed that bulliform cell numbers between two vascular bundles in *mOsARF18* leaves were significantly reduced when compared with that of wild type (Fig. 4i). Numbers of total vascular bundles and abaxial epidermal cells between two vascular bundles were also decreased (Fig. 4d,g,h,j,k). In addition, with the exception of the bulliform cells, we did not observe significant size differences in other cells when comparing *mOsARF18* leaves with the wild-type. Thus, our results suggest that repression of *OsARF18* by *Os*miR160 is important for epidermal cell division and bulliform cell differentiation during leaf development.

### Starch accumulation during seed development was abnormal in *mOsARF18* plants

To study why *mOsARF18* plants produced small seeds, we examined starch accumulation in developing seeds. Our results demonstrated that endosperm in wild-type developing seeds contained many starch granules that were strongly stained by iodine (Fig. 4l), whereas endosperm in *mOsARF18* developing seeds contained smaller, weakly stained starch granules (Fig. 4m,n). In conclusion, *mOsARF18* plants were defective in starch accumulation during seed development.

### The *Os*miR160-regulated *OsARF18* may control rice growth and development via affecting auxin signaling

**Figure 5:**
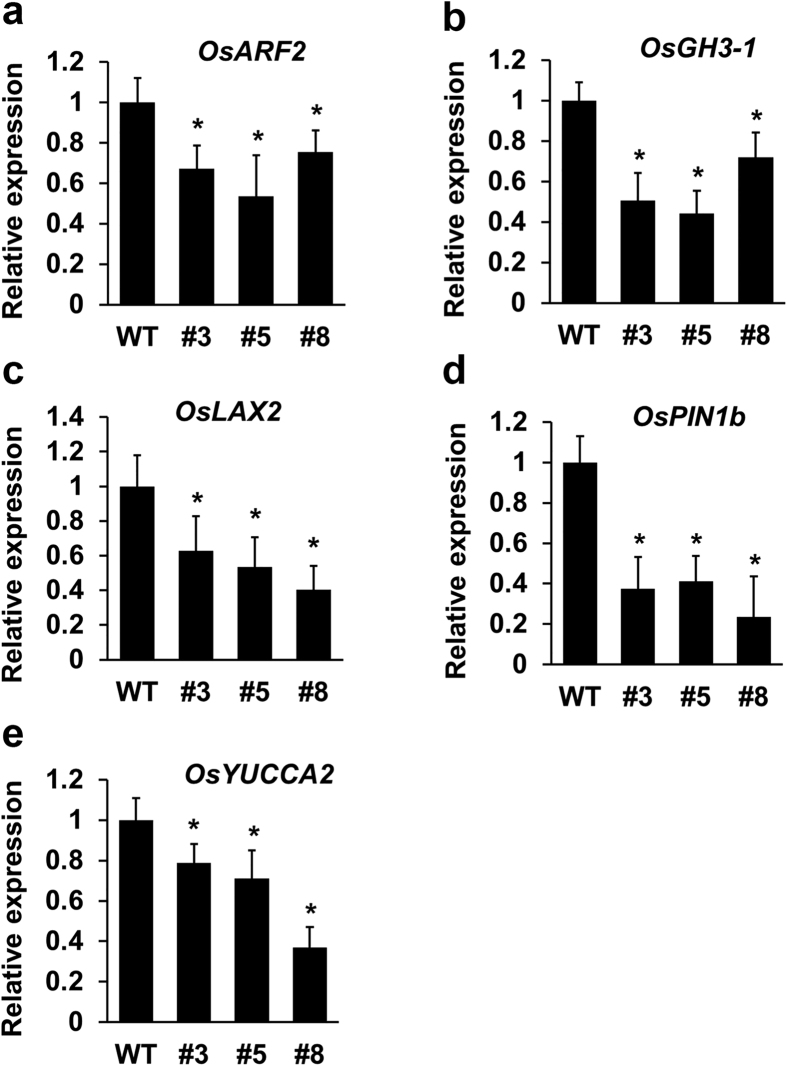
Analysis of auxin signaling gene expression in *mOsARF18* transgenic plants. qRT-PCR results showing that expression of *OsARF2* (**a**), *OsGH3-1* (**b**), *OsLAX2* (**c**), *OsPIN1b* (**d**), and *OsYUCCA2* (**e**) was significantly decreased in leaves of *mOsARF18* transgenic plants. Gene expression levels in *mOsARF18-3*, *mOsARF18-5*, and *mOsARF18-8* were normalized based on expression observed in wild type. Stars indicate significant difference (P < 0.01).

ARFs play a primary role in auxin signaling. To test whether auxin signaling was affected in *mOsARF18* transgenic plants, we examined expression of representative genes known to be involved in auxin signaling in rice, including the *AUXIN RESPONSIVE FACTOR* gene *OsARF2*^34^, the auxin responsive gene *OsGH3-1*^35^, the *AUX1-LIKE* gene *OsLAX2* (*OsAUX3* or *OsRAU2; Os*03g14080)^36^, the auxin efflux gene *OsPIN1b*^37^, and the auxin biosynthesis gene *OsYUCCA2*^38^ via qRT-PCR. Expression levels of all tested genes were significantly decreased in *mOsARF18* transgenic plant leaves (Fig. 5a–e). Thus, our results suggest that deregulation of *OsARF18* affects auxin signaling, which might cause abnormal growth and development in rice.

**Figure 6:**
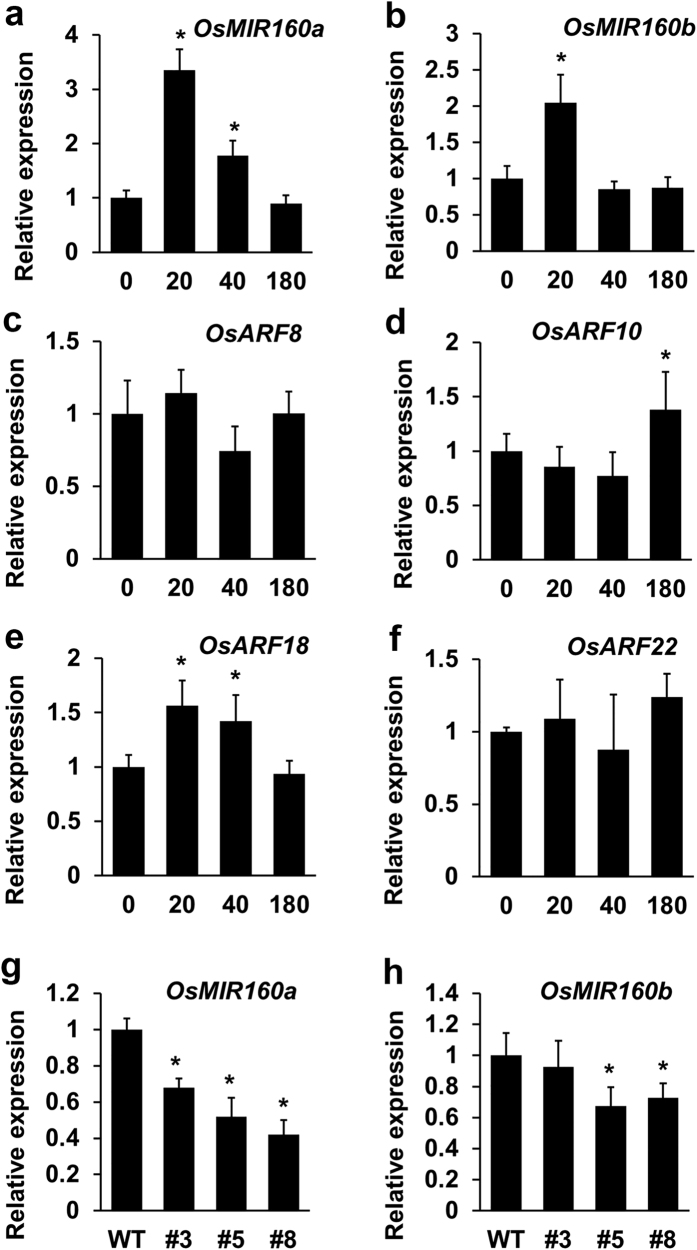
Auxin up regulated expression of *OsMIR160a, OsMIR160b*, and *Os*miR160 target genes, whereas *Os*ARF18 suppressed the expression of *OsMIR160a*. (**a–f**) qRT-PCR results showing that expression changes of *OsMIR160a*, *OsMIR160b*, and *Os*miR160 target genes in seedlings (7-day old) treated with NAA for 20, 40, and 180 minutes. Expression levels without NAA treatment (0 minute) were used to normalize expression with treatments. Stars indicate significant difference (P < 0.01). Expression of *OsMIR160a* was significantly induced after 20 and 40-minute treatment (**a**), while expression of *OsMIR160b* was significantly increased only after 20-minute treatment (**b**). Expression of *OsARF8* (**c**) and *OsARF22* (**f**) remained unchanged. Expression of *OsARF10* was significantly induced after 180-minute treatment (**d**), whereas expression of *OsARF18* was significantly increased after 20 and 40-minute treatment (**e**). (**g,h**) qRT-PCR results showing that expression of *OsMIR160a* and *OsMIR160b* was significantly decreased in *mOsARF18* transgenic lines in comparison with wild-type plants. Gene expression levels in #3 (*mOsARF18-3*), #5 (*mOsARF18-5*), and #8 (*mOsARF18-8*) were normalized based on expression observed in wild type. Stars indicate significant difference (P < 0.01).

We further tested whether auxin affected expression of *OsMIR160a*, *OsMIR160b*, and *Os*miR160 target genes. Our qRT-PCR results showed that expression of *OsMIR160a* was significantly increased after 20 and 40-minute NAA treatment (Fig. 6a), while expression of *OsMIR160b* was significantly induced after 20-minute NAA treatment and then remained normal (Fig. 6b). NAA treatment also significantly induced expression of *OsARF18* after 20 and 40 minutes (Fig. 6e) as well as expression of *OsARF10* after 180 minutes (Fig. 6d). However, expression of *OsARF8* (Fig. 6c) and *OsARF22* (Fig. 6f) remained unchanged after NAA treatment.

Conversely, expression of *OsMIR160a* and *OsMIR160b* was found to be decreased in *mOsARF18* plants (Fig. 6g,h), in which the expression of *OsARF18* was highly increased (Fig. 2b). Our results suggest that auxin up regulates expression of *OsMIR160a*, *OsMIR160b*, and *OsARF18*, whereas *Os*ARF18 represses expression of *OsMIR160a* and *OsMIR160b*. Therefore, the positive regulation of *OsMIR160a* and *OsMIR160b* expression by auxin and the negative regulation of *OsMIR160a* and *OsMIR160b* by *Os*ARF18 might be important for *Os*miR160 to fine-tune auxin signaling in a negative feedback loop manner.

**Figure 7:**
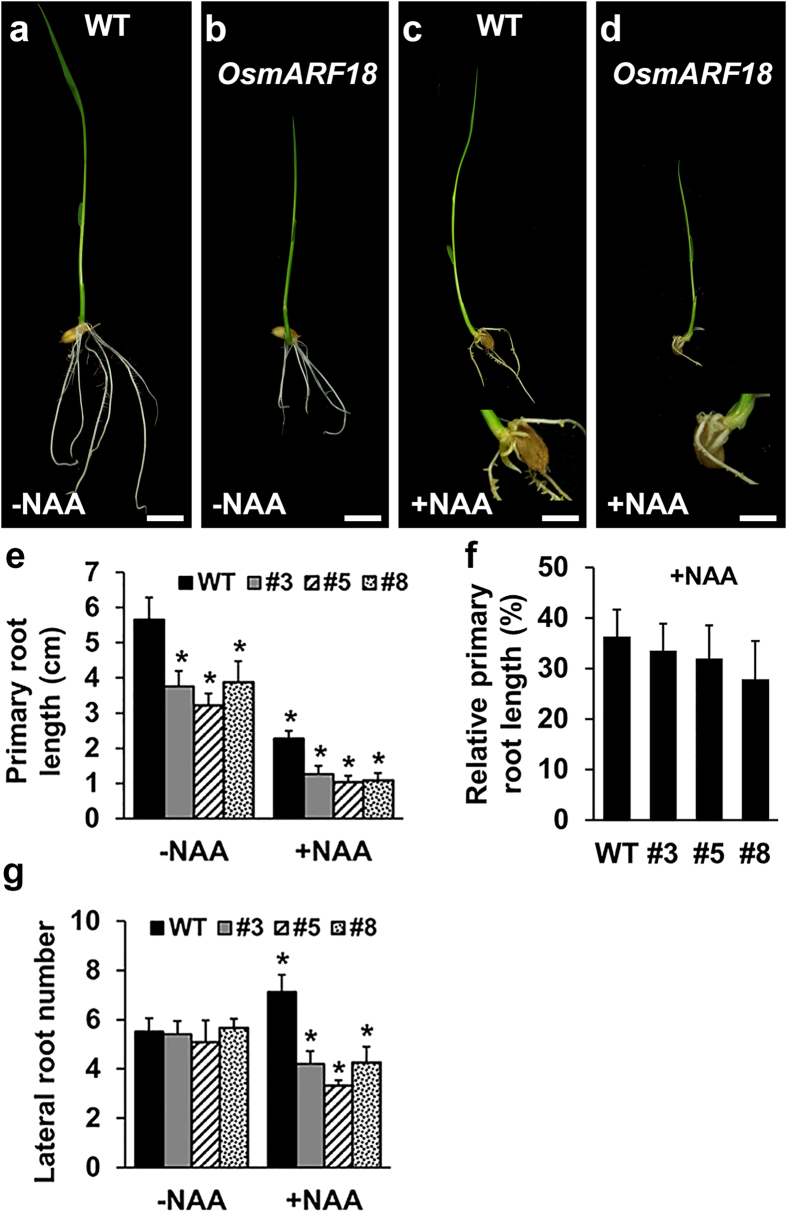
Effect of NAA treatment on lateral root formation. (**a,c**) Seven-day wild type (WT) seedlings without NAA (**a**) and with NAA (1 μM) (**c**) treatment (inset showing high magnification of roots in (**c**). (**b,d**) Seven -day *OsmARF18* seedlings without NAA (**b**) and with NAA (1 μM) (**d**) treatment (inset showing high magnification of roots in (**d**). (**e,f**) Primary roots in WT 7-day seedlings were significantly (*indicating P < 0.01) longer than that of *OsmARF18* seedlings. NAA treatment significantly (P < 0.01) inhibited primary root length of both WT and *OsmARF18* seedlings, but the relative primary root length (percentage of root length between that treated and untreated) was similar. (**g**) Seven-day WT and *OsmARF18* seedlings produced similar numbers of lateral roots. After NAA treatment, lateral root numbers in WT seedlings was significantly (P < 0.01) increased, whereas that in *OsmARF18* seedlings was significantly (P < 0.01) decreased.

To test whether *OsmARF18* seedlings are defective in auxin signaling, we treated *OsmARF18* seedlings hydroponically with 1 μM of NAA for 7 days. We found that *OsmARF18* seedlings produced significantly shorter primary roots than that of wild type (Fig. 7a,b,e). Primary root growth was significantly inhibited by NAA treatment in both wild type and *OsmARF18* seedlings (Fig. 7c–e); however, the NAA inhibition effect on primary root length was similar in wild type and *OsmARF18* seedlings, because there was no change in relative primary root length (percentage of root length between that treated and untreated) after the NAA treatment (Fig. 7f). Numbers of lateral roots were similar in wild type and *OsmARF18* seedlings (Fig. 7a,b,g). NAA treatment significantly increased the lateral root number in wild type seedlings (Fig. 7a,c,g), conversely, the lateral root number in *OsmARF18* seedlings was significantly decreased with the NAA treatment (Fig. 7b,d,g). Our results suggest that *OsARF18* might be involved in auxin-regulated lateral root formation in rice.

## Discussion

### Conserved and diverse roles of miR160 in plant growth and development

MiR160 is conserved throughout the plant kingdom from mosses to higher plants^29^,^30^. Sequence similarity of mature miR160s is more than 80% among different species. MiR160 target genes are also conserved, although their numbers vary with species. *Arabidopsis* contains *MIR160a*, *MIR160b*, and *MIR160c* three genes which produce the same mature miR160^6^. MiR160 targets *AUXIN RESPONSE FACTOR 10* (*ARF10*), *ARF16*, and *ARF17* three genes and each target gene has conserved but some distinct functions in *Arabidopsis*. Plants expressing the miR160-resistant version of *ARF10* (m*ARF10*) exhibit pleiotropic defects that resemble phenotypes of some ABA and auxin defective mutants^24^, while *mARF16* plants have reduced fertility and less lateral roots^22^. ARF10 and ARF16 are required for maintaining the expression of *ABI3* gene^25^, suggesting that ARF10 and ARF16 are involved in both auxin and ABA signaling or cross-talk between them. ARF17 plays a general role in both vegetative and reproductive development via modulating expression of early auxin response genes^23^. *Sly*miR160 and the *Sly*miR160a target *SlyARF10* control ovary patterning, early fruit development and floral organ abscission in tomato^26^,^27^, whereas the soybean miR160 regulates auxin and cytokinin signaling during nodulation^28^.

Six *OsMIR160* (*OsMIR160a* to *OsMIR160f*) genes are found in rice. The mature *Os*miR160 has *OsARF8* (*Os*02g41800), *OsARF10* (Os04g43910), *OsARF18* (*Os*06g47150), and *OsARF22* (*Os*10g33940) four potential target genes. In this study, we characterized the function of *Os*ARF18 (*Os*06g47150), an orthologue of *Arabidopsis* ARF16. *Arabidopsis mARF16* and rice *mOsARF18* plants show some similar but also different defects in growth and reproductive development. In *Arabidopsis*, *mARF16* plants produce curved leaves^22^. Our studies showed that the formation of rolled leaves in *mOsARF18* plants was caused by alterations in shape, size, and number of bulliform cells. Auxin plays a key role in controlling leaf shape^39^. Phenotypic analysis indicates that alteration of bulliform cells on the adaxial leaf blade surface is tightly linked to the formation of rolled leaves in rice. Disruption of the rice *CONSTITUTIVELY WILTED1* (*OsCOW1*) gene, a member of *OsYUCCA* gene family, results in rupture of the largest bulliform cell and consequently rolled leaves^40^. In addition, alterations in number and size of bulliform cells lead to rolled leaves in the *narrow leaf 7* (*nal7*) mutant, which is an *oscow1* allele^41^. Therefore, our data support that the *Os*miR160 target *OsARF18* controls leaf shape via affecting auxin signaling. Besides rolled leaves, *mOsARF18* plants produced less and smaller seeds than wild type. Moreover, starch accumulation in seeds from *mOsARF18* plants was significantly decreased when compared to wild-type seeds. Collectively, miR160 plays conserved and diverse roles in plant growth and development. It will be interesting to test the loss-of-function of *Os*miR160 and functions of other *Os*miR160 target genes in rice growth and development.

### Negative regulation of *OsARF18* by *Os*miR160 is important for its normal function

MiRNAs negatively regulate gene expression at the post-transcriptional level by binding to mRNA complementary sequences for mRNA destabilization and translational inhibition in both plants and animals^1^,^2^,^3^,^4^,^5^. One miRNA normally has multiple target genes. Over/ectopic-expression of normal miRNA-target genes usually does not cause a change in phenotype, because over/ectopic-expressed normal mRNAs can be still targeted by miRNAs for cleavage. Therefore, a primary approach for studying the function of miRNA and its target is to express the miRNA-resistant version of individual target gene. Employing *35S*, *Ubi*, and *ACTIN* promoters, previous studies identified functions of many miRNAs and their target genes via expressing miRNA-resistant versions of target genes, such as *35S::mTCP2*, *35S::mTCP3*, *35S::mTCP4*, *35S::mCUP1*, and *35S::mCUP2* (*Arabidopsis* miR164 target genes)^42^,^43^, *35S::mAP2* (*Arabidopsis* miR172 target gene)^44^, *35S::mSlyARF10* (tomato miR160 target gene)^26^, *UBI:mGRF6* (rice miR396 traget gene)^45^, as well as *ACTIN*::*mOSHB1*, *ACTIN*::*mOSHB3* and *ACTIN*:: *mOSHB5* (rice miR166 target genes)^46^.

So far, no studies report that over/ectopic-expression of normal versions of miR160 target genes (*ARF10*, *ARF16*, and *ARF17*) causes phenotypes in leaf and flower development, but only plants expressing miR160-resistant versions of miR160 target genes (*mARF10*, *mARF16*, and *mARF17*) exhibit various phenotypes^22^,^23^,^24^,^26^. In this study, we used the *Ubiquitin* (*Ubi*) promoter to drive *mOsARF18* expression. To further rule out the possibility that the phenotype of *mOsARF18* was caused by ectopic activity of the *Ubi* promoter, we generated *ARF16*, *mARF16*, *OsARF18*, and *mOsARF18 Arabidopsis* transgenic plants under the control of the *Ubi* promoter. Of 30 *ARF16* and 32 *OsARF18* transgenic plants that we obtained, none of them showed detectable defects in growth and development (Fig. S5a–c,f–h; Fig. S6a–c,f–h,k–m,p); however, *mARF16* (32 out of 40) and *mOsARF18* (20 out of 29) transgenic plants formed narrow and curled leaves, and had short stature (Fig. S5d,e,i,j). In addition, *mARF16* and *mOsARF18* plants produced abnormal flowers and a lower number of smaller seeds compared with wild-type plants (Fig. S6d,e,i,j,n,o,q). Our results showed that *mARF16* and *mOsARF18* caused defects in plant growth and development, but plants ectopically expressing normal *ARF16* and *OsARF18* were similar to the wild type. Taken together, the results from *Arabidopsis* and rice suggest that the negative regulation of *OsARF18* and its *Arabidopsis* orthologous gene *ARF16* by rice and *Arabidopsis* miR160, respectively, is critical for their function.

### Molecular mechanism of miR160 in fine-tuning auxin signaling

MiRNAs play a pivotal role in auxin signaling by negative regulation of *ARFs*. In *Arabidopsis*, miRNAs are involved in expression regulation of 8 of a total 23 *ARFs* (*ARF2*, *3*, *4*, *6*, *8*, *10*, *16*, and *17*). MiR390-derived trans-acting-small interfering RNAs (ta-siRNAs) target *ARF2*, *ARF3*, and *ARF4*^47^,^48^. The tasiRNA gradient is important for establishing the normal patterning of ARF3 protein during leaf development^49^. During growth of lateral roots, miR390 affects production of tasiRNAs, and thus inhibits *ARF2*, *ARF3*, and *ARF4*^50^. Conversely, auxin activates ARF2, ARF3, and ARF4, which consequently influences the formation of miR390. Therefore, the regulatory network modulated by miR390 maintains normal expression of *ARF2*, *ARF3*, and *ARF4*. Negative regulation of *ARF6* and *ARF8* by miR167 is essential for anther and ovule development^51^. Moreover, miR160 negatively regulates expression of *ARF10*, *ARF16*, and *ARF17*^6^,^22^,^23^,^24^. In rice, *Os*miRNAs are predicted to regulate 11 of a total of 25 identified *OsARFs*^52^,^53^. Functional disruption of *OsDCL4* causes increased expression of *Os*miR165/166 and three *OsARFs*, which are orthologs of *Arabidopsis ARF2*, *ARF3*, and *ARF4*^54^. *Os*miR160 is predicted to have four *OsARF* targets, including *OsARF18* that was characterized in this study. *Os*miR167 potentially targets *OsARF6* (*Os*02g06910), *OsARF12* (*Os*04g57610), *OsARF17* (*Os*06g46410), and *OsARF25* (*Os*12g41950), which are highly similar to *Arabidopsis ARF6* and *ARF8*^54^. Together, miRNAs target a group of similar *ARF* genes in both monocots and dicots.

Auxin promotes the SCF^TIR1/AFB^ E3 ligase-mediated degradation of Aux/IAA proteins, which sequester ARFs^55^,^56^. Upon the perception of auxin, the released ARFs activate or suppress expression of a large set of auxin-responsive genes by binding to auxin response elements (AuxREs) in their regulatory regions. Based on transient expression studies and protein structures, among 23 ARFs in *Arabidopsis*, ARF5-8, and 19 act as activators, while ARF1-4, 9-18, and 20-22 may function as repressors^57^,^58^,^59^.

Previous studies have implied that the feedback regulation between miRNA and its target *ARF* genes could provide a fine-tuning mechanism to regulate auxin signaling. During adventitious root development in *Arabidopsis*, expression of *ARF6* and *ARF8* is regulated by miR167 and miR160, whereas the abundance of *ARF17* transcripts is controlled by miR160^60^. In addition, ARF6 and ARF8 activators as well as ARF17 repressors positively and negatively regulate the expression of each other. Thus, the delicate balance between miRNAs and *ARFs* is critical for auxin-regulated developmental processes. Our studies show that *Os*miR160 negatively regulates the expression of *OsARF18* by cleaving *OsARF18* transcripts. Auxin induces expression of *OsMIR160a*, *OsMIR160b*, and *OsARF18*, whereas expression of *OsMIR160a* and *OsMIR160b* was suppressed by *Os*ARF18. Our analyses revealed that promoters and 3′ regions of *OsMIR160a* and *OsMIR160b* had clusters of AuxRE cores and AuxRR *cis* elements (Fig. S4), which may create *Os*ARF binding sites by which *Os*ARF activators or repressors could regulate expression of *OsMIR160a* and *OsMIR160b*.

**Figure 8:**
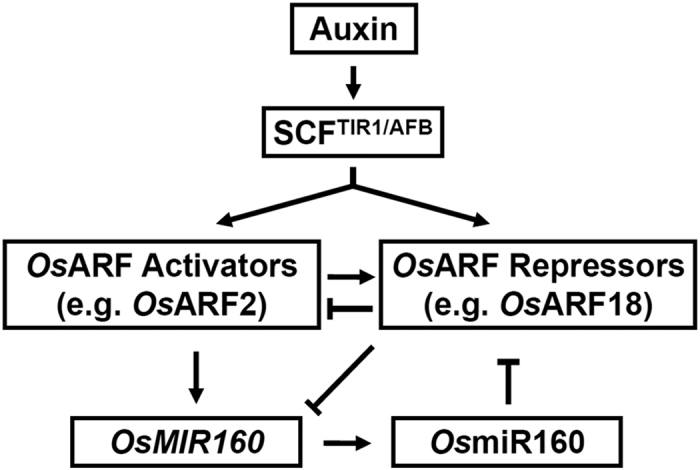
A hypothetical working model for auxin signaling that is modulated by *Os*miR160 during rice growth and development. Auxin promotes the release of *Os*ARF activators or repressors via the auxin receptor SCF E3 ligase. The balance between *Os*ARF activators and repressors decides up or down expression of *OsMIR160* genes. Conversely, the change in abundance of mature *Os*miR160 negatively regulates expression of its target *OsARFs*. *Os*ARF activators and repressors might positively or negatively regulate expression of each other.

In our hypothetical model, auxin promotes the release of *Os*ARF activators and repressors via the SCF^TIR1/AFB^ E3 ligase (Fig. 8). The balance between *Os*ARF activators and repressors decides up or down expression of *OsMIR160* genes, which consequently affects the abundance of mature *Os*miR160. Conversely, *Os*miR160 negatively modulates expression of its target *OsARF*s. *OsARF*s also mutually control their own expression. Future studies should focus on examining the feedback loop regulation between *Os*miR160 and its target *Os*ARFs, which might be important for fine-tuning highly dose-sensitive auxin signaling during rice growth and development.

Manipulating miRNAs and their target genes has demonstrated improvement of many important crop traits, including biomass yield, grain yield, fruit yield, nutritional quality, abiotic stress resistance (e.g. drought, salinity, cold, heat, oxidative stress, nutrient deprivation tolerance, and heavy metal detoxification), and biotic stress resistance (e.g. virus, bacteria, fungus, and nematode resistance)^14^,^15^,^61^,^62^. Our results showed that *Os*miR160 played a pivotal role in rice growth and development via regulating auxin signaling. In particular, *Os*miR160 is essential for leaf and seed development in rice. Leaf shape is important for photosynthesis, respiration, and transpiration. Moderate leaf rolling can enhance photosynthesis and stress responses by inhibiting water loss and radiant heat absorption, which, therefore, increases crop yield. Future studies on the molecular mechanism by which *Os*miR160 modulates auxin signaling will lead to potential applications for improving crop agronomic traits.

## Methods

### Plant materials and growth condition

Rice (*Oryza sativa* L. Japonica Nipponbare) plants were grown in Metro-Mix 360 soil (Sun-Gro Horticulture, Agawam, MA, USA) supplemented with sand and iron in a walk-in growth chamber under a 12-hour light (28 °C)/12-hour dark (22 °C) photoperiod regime. Transgenic rice plants were generated at the Plant Transformation Facility at Iowa State University. In total, 124 *mOsARF18* plants were obtained from 19 independent transgenic lines. For expression studies, seven-day old wild-type seedlings were treated with 1 μm of NAA for 20, 40, and 180 min with untreated 7-day old seedlings as control. For studying the effect of exogenous auxin on root development, geminated seeds were hydroponically grown in the 1/2 Kimura B nutrient solution (pH 5.6) containing 1 μm of NAA for 7 days with continuous light at 25 °C.

### Vector construction and rice transformation

PCR reactions (Primers are shown in Table S1) were performed using the Phusion High-Fidelity DNA Polymerase (New England Biolabs, Ipswich, MA, USA). The *OsARF18* cDNA was amplified from rice leaf cDNAs and then cloned into the pCR2.1 vector (Invitrogen, Grand Island, NY, USA), resulting in *pCR2.1*-*OsARF18*. Point mutations of *OsARF18* were created by overlapping PCR to generate *pCR2.1*-*mOsARF18*. *mOsARF18* was then subcloned into the modified pCambia1301 binary vector harboring the Gateway cassette sequence and the maize *Ubiquitin* (*Ubi*) promoter using the Gateway LR recombinase II enzyme mix (Invitrogen, Grand Island, NY, USA).

For rice transformation, the *mOsARF18* construct was introduced into the *Agrobacterium* strain EHA101. The callus induction (from mature embryos of Japonica cv. Nipponbare seeds), *Agrobacterium* infection, co-cultivation, selection of transformed calli, and plant regeneration were performed essentially as described previously^63^.

### RT-PCR and real time qRT-PCR

Total RNAs were isolated from different rice tissues using the RNeasy Plant Mini Kit (Qiagen, Valencia, CA, USA). After determining the RNA quantification by the NanoDrop 2000c (Thermo Scientific, Bannockburn, IL, USA), RNA reverse transcription was conducted using the QuantiTect Reverse Transcription Kit (Qiagen, Valencia, CA, USA). Real-time PCR (DNA Engine Opticon 2 system, Hercules, California, USA) and data analysis were performed as previously described^6^. Expression of *OsMIR160a* through *OsMIR160f* was examined by RT-PCR. Expression of *OsMIR160a*, *OsMIR160b*, *OsARF8, OsARF10*, *OsARF18*, and *OsARF22*, as well as other auxin signaling related genes was tested by real-time qRT-PCR. Three biological repeats were conducted and each value indicates the average with the standard error. All primer sequences are shown in Table S1.

### 5′ RACE

Using the GeneRacer^TM^ kit (Invitrogen, Grand Island, NY, USA), a gene-specific 5′-rapid amplification of cDNA ends was conducted as described previously^23^. Gene-specific primers for *OsARF18* (*Os*06g47150) are shown in Table S1.

### Semi-thin section analysis

Semi-thin sectioning was performed as described previously^64^,^65^. The fifth leaves of 6-week-old rice were fixed in 2.5% of glutaraldehyde and post-fixed with 1% of OsO4 at room temperature. Samples were dehydrated through a graded acetone series (10% increments) for 60 minutes each. Infiltrated was started with 20% of Spurr’s resin and then 40%, 60%, and 80% of Spurr’s resin every 3 hours. Following infiltration in three changes of 100% Spurr’s resin for 24 hours each, samples were finally embedded in 100% Spurr’s resin and polymerized at 60 °C overnight. Semi-thin sections (0.5 μm) were made using an RMC MT-7 ultramicrotome (Reichert-Jung, Depew, NY, USA) and were stained with 0.25% Toluidine Blue O. Images were photographed by an Olympus BX51 microscope equipped with an Olympus DP 70 digital camera (Olympus, Center Valley, PA, USA).

### Histological detection of starch

Ten-DAP (Days After Pollination) seeds were fixed in FAA (50% ethanol, 10% formalin, 5% acetic acid). Following fixation, samples were dehydrated through an ethanol series, embedded in paraffin, and sectioned at 8 μm with a Spencer 820 microtome. Sections were dewaxed and stained with Lugol’s iodine solution (6 mM iodine, 43 mM KI, and 0.2 N HCl) for detection of starch granules. Images were photographed with an Olympus BX51 microscope equipped with an Olympus DP 70 digital camera (Olympus, Center Valley, PA, USA).

## Additional Information

**How to cite this article**: Huang, J. *et al*. Deregulation of the *Os*miR160 Target Gene *OsARF18* Causes Growth and Developmental Defects with an Alteration of Auxin Signaling in Rice. *Sci. Rep*. **6**, 29938; doi: 10.1038/srep29938 (2016).

## Supplementary Material

Supplementary Information
