# Supplementary material for: Deregulation of the OsmiR160 Target Gene OsARF18 Causes Growth and Developmental Defects with an Alteration of Auxin Signaling in Rice
Source: Sci Rep. 2016 Jul 21;6:29938. doi: 10.1038/srep29938 (PMC4956771; doi:10.1038/srep29938)
Supplement: Supplementary Information [file srep29938-s1.pdf]

Supplementary information

Deregulation of the *Os*miR160 Target Gene *OsARF18* Causes Growth and Developmental Defects with an Alteration of Auxin Signaling in Rice

Jian Huang, Zhiyong Li and Dazhong Zhao
Department of Biological Sciences, University of Wisconsin-Milwaukee, Milwaukee, WI 53211, USA

Methods

Vector construction and transformation.

PCR reactions (Primers are shown in Table S1) were performed using the Phusion High-Fidelity DNA Polymerase (New England Biolabs, Ipswich, MA, USA). The *OsARF18* cDNA was amplified from rice leaf cDNAs. *OsARF18* and *ARF16* (from the U11294 plasmid) cDNAs were cloned into the pCR2.1 vector (Invitrogen, Grand Island, NY, USA), resulting in *pCR2.1-OsARF18* and *pCR2.1-ARF16*. Point mutations of *OsARF18* and *ARF16* were performed by overlapping PCR to generate *pCR2.1-mOsARF18* and *pCR2.1-mARF16*. *OsARF18, mOsARF18, ARF16,* and *mARF16* were then subcloned into the pGCS binary vector harboring the *Ubiquitin* (*Ubi*) promoter to generate *pGCS-OsARF18, pGCS-mOsARF18, pGCS-ARF16,* and *pGCS-mARF16* constructs using the Gateway LR recombinase II enzyme mix (Invitrogen, Grand Island, NY, USA).

For *Arabidopsis* transformation, *pGCS-OsARF18, pGCS-mOsARF18, pGCS-ARF16,* and *pGCS-mARF16* were transformed into the *Agrobacterium* strain GV3101. The floral dip method was used to generate transgenic *Arabidopsis* plants[1]. Transformants were screened on ½ Murashige and Skoog (MS) plates containing 25 μg/mL of hygromycin.

Reference

1. Clough, S.J. & Bent A.F. Floral dip: a simplified method for Agrobacterium-mediated transformation of *Arabidopsis thaliana*. *Plant J.* **16**, 735-743 (1998).

Figures and figure legends

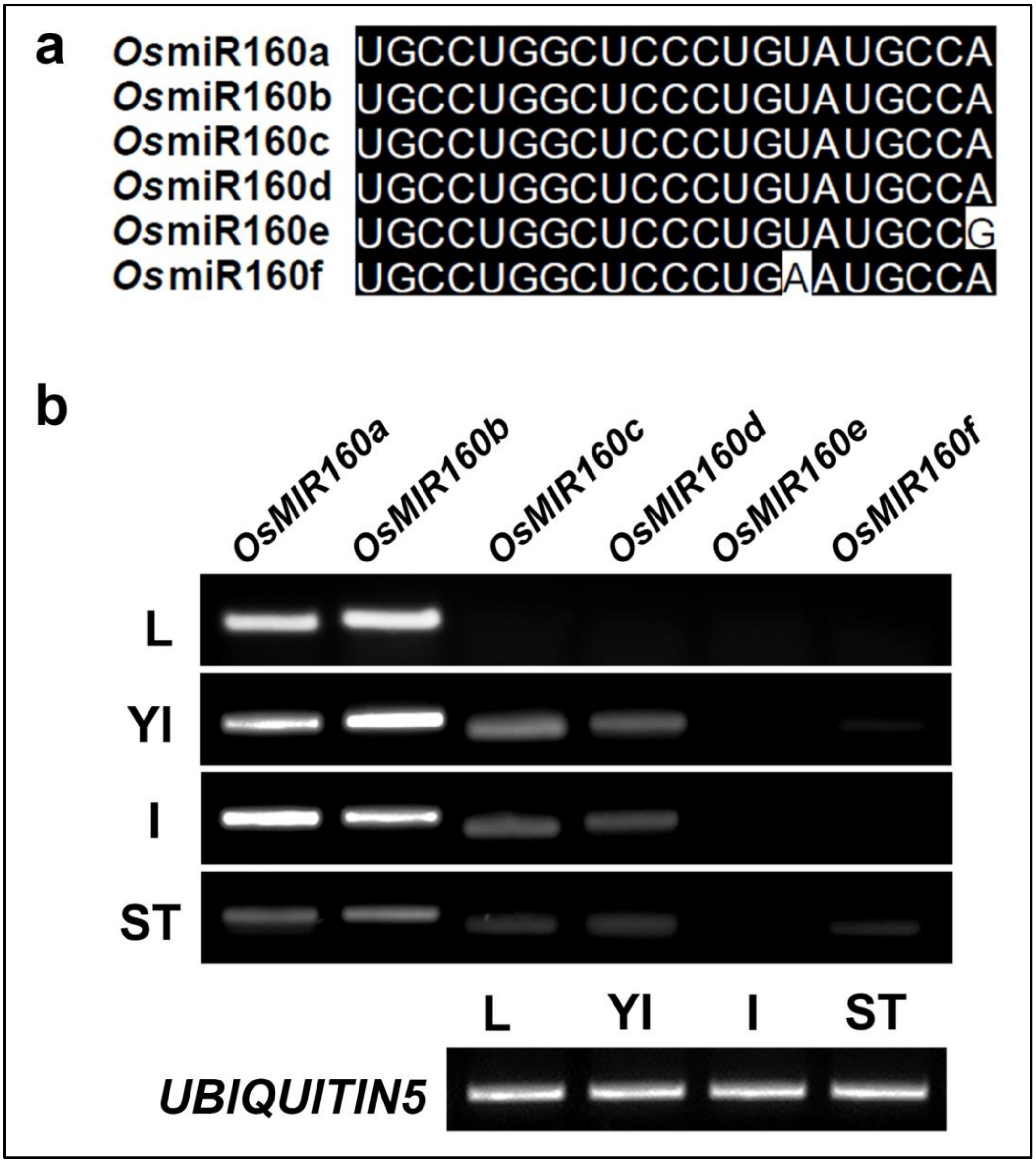

Figure S1. Alignment of mature *Os*miR160s in rice as well as *OsMIR160a* and *OsMIR160b* genes were dominantly expressed in leaf.

(**a**) Sequence alignment of six mature *Os*miR160s in rice. (**b**) RT-PCR results showing expression of six *OsMIR160* genes in leaf (L), young inflorescence (YI), mature inflorescence (MI), and stem (ST).

```
AtARF10             -----------ME--QEKSLDPQLWHACAGSMVQIPSLNSTVFYFAQGHTEHAHAPP--- 44
AtARF16             ----MINVMNPMKGGTEKGLDPQLWHACAGGMVRMPPMNSKVFYFPQGHAENAYDCV--- 53
OsARF18_Os06g47150  MITFVDSAAKERERESDKCLDPQLWHACAGGMVQMPPVSSKVYYFPQGHAEHAQGHG--- 57
OsARF22_Os10g33940  -------MKEVGEVEEVRCLDPQLWHACAGGMVQMPAPRSRVYYFAQGHAEHADGGGGAA 53
OsARF8_Os02g41800   -MITFADLAEPAPG-AERCVDRQLWLACAGGMCTVPPVGAAVYYFPQGHAEHALGLAAP- 57
OsARF10_Os04g43910  -MLTFMELAGPTEGDGGGSVDSQLWAACAGSMSSVPPVGAAVYYFPQGHAEQAS--AAV- 56
AtARF17             ----MSPPSATAGDINHREVDPTIWRACAGASVQIPVLHSRVYYFPQGHVEHCCPLLS-- 54
                                       :*  :* ****.   :*   : *:**.***.*:.

AtARF10             ----DFHAPRVPPLILCRVVSVKFLADAETDEVFAKITLLPLPGNDLDLENDAVLGLTPP 100
AtARF16             ----DFGNLPIPPMVLCRVLAIKYMADAESDEVFAKLRLIPLK-DDEYVDHEYGDGED-- 106
OsARF18_Os06g47150  --PVEFPGGRVPALVLCRVAGVRFMADPDTDEVFAKIRLVPVRANEQGYAG-DADDGIGA 114
OsARF22_Os10g33940  AAAAELGPRALPPLVLCRVEGVQFLADRDSDEVYAKIRLAPVAPGEAEFREPDELCPLGA 113
OsARF8_Os02g41800   ----ELSAARVPALVPCRVASVRYMADPDTDEVFARIRLVPLRAAEDG-DVEEDGAAAG- 111
OsARF10_Os04g43910  ----DLSSARVPPLVPCRVVAVRFMADAESDEVFAKIRLVPLRPGDAVVDVGEAAAAEAR 112
AtARF17             ----TLPSSTSP--VPCIITSIQLLADPVTDEVFAHLILQPMTQQQFTPTNYSRFGRFDG 108
                         :     *  : * : .:: :**  :***:*:: * *:   :

AtARF10             SSDGNGNGKEKPASFAKTLTQSDANNGGGFSVPRYCAETIFPRLDYSAEPPVQTVIAKDI 160
AtARF16             -SNGFESNSEKTPSFAKTLTQSDANNGGGFSVPRYCAETIFPRLDYNAEPPVQTILAKDV 165
OsARF18_Os06g47150  AAAAAAQE-EKPASFAKTLTQSDANNGGGFSVPRYCAETIFPRLDYSADPPVQTVLAKDV 173
OsARF22_Os10g33940  AGDAAEPSPEKPTSFAKTLTQSDANNGGGFSVPRYCAETIFPKLDYRADPPVQTVLAKDV 173
OsARF8_Os02g41800   ----EEHEKP--ASFAKTLTQSDANNGGGFSVPRYCAETIFPRLDYAADPPVQTVVAKDV 165
OsARF10_Os04g43910  --REEENSRPRPTSFAKTLTQSDANNGGGFSVPRFCAETIFPELDYSSEPPVQSVCAKDV 170
AtARF17             ----DVDDNNKVTTFAKILTPSDANNGGGFSVPRFCADSVFPLLNFQIDPPVQKLYVTDI 164
                                .:*** ** *************:**:::** *::  :****.: ..*:

AtARF10             HGETWKFRHIYRGTPRRHLLTTGWSTFVNQKKLIAGDSIVFLRSESGDLCVGIRRAKRGG 220
AtARF16             HGDVWKFRHIYRGTPRRHLLTTGWSNFVNQKKLVAGDSIVFMRAENGDLCVGIRRAKRGG 225
OsARF18_Os06g47150  HGVVWKFRHIYRGTPRRHLLTTGWSTFVNQKKLVAGDSIVFMRTENGDLCVGIRRAKKGG 233
OsARF22_Os10g33940  HGVVWKFRHIYRGTPRRHLLTTGWSTFVNQKKLVAGDSIVFLRTRHGELCVGIRRAKRMA 233
OsARF8_Os02g41800   HGVAWNFRHIYRGTPRRHLLTTGWSTFVNQKKLVAGDSIVFLRGDGGDLHVGIRRAKRGF 225
OsARF10_Os04g43910  HGVEWTFRHIYRGTPRRHLLTTGWSPFVNKKQLTAGDSIVFMRDEGGNIHVGLRRAKRGF 230
AtARF17             HGAVWDFRHIYRGTPRRHLLTTGWSKFVNSKKLIAGDSVVFMRKSADEMFIGVRRTPISS 224
                    **  * ******************* ***.*:* ****:**:*    .:: :*:**:

AtARF10             LGSN---AG------SDNPYP---GFSGFLRDDESTTTTSKLMMMKRNGNNDGNAAATGR 268
AtARF16             IGNGPEYSA------GWNPIGGSCGYSSLLREDESNS-------LRRS--NCSLADRKGK 270
OsARF18_Os06g47150  VGGPEFLPPPPPPPPTPAAGGNYGGFSMFLRGDDDGN-------KMAAAARG---KVRAR 283
OsARF22_Os10g33940  CGGMECMSG----WNAPGYGG--GGFSAFLK-EEESK-------LMKGHGGGGYMKGKGK 279
OsARF8_Os02g41800   CGGGG--GA-----EEAS-LPGWDQYGGLMRGNASPC-----------AAAK---GRGK 262
OsARF10_Os04g43910  CSIGG--DD-----ESLSSIPGWDQYRGLMRRNATAT------------ATGGRTPPKGK 271
AtARF17             SDGGSSYYG-------GDEYNGYYSQSSVAKEDDGSP------------KKTFRRSGNGK 265
                     .                          . : :                         .:

AtARF10             VRVEAVAEAVARAACGQAFEVVYYPRASTPEFCVKAADVRSAMRIRWCSGMRFKMAFETE 328
AtARF16             VTAESVIEAATLAISGRPFEVVYYPRASTSEFCVKALDARAAMRIPWCSGMRFKMAFETE 330
OsARF18_Os06g47150  VRPEEVVEAANLAVSGQPFEVVYYPRASTPEFCVKAGAVRAAMRTQWFAGMRFKMAFETE 343
OsARF22_Os10g33940  VRMADVVEAASLASSGQPFEVAYYPRASTPDFVVKAASVQAAMRIQWCSGMRFKMAFETE 339
OsARF8_Os02g41800   VRAEDLVEAARLANGGQPFEVVYYPRASTPEFCVRAAAVRAAMRVQWCPGMRFKMAFETE 322
OsARF10_Os04g43910  VPPENVLTAATRATTGQPFEVLYYPRASTPEFCVRAAAVRTAMAVQWCPGMRFKMAFETE 331
AtARF17             LTAEAVTDAINRASQGLPFEVVFYPAAGWSEFVVRAEDVESSMSMYWTPGTRVKMAMETE 325
                    :    :  *    *   * .*** :** *.  .:* *:*   ..::*    * .* *.***:***

AtARF10             DSSRISWFMGTVSAVQVADPIRWPNSPWRLLQVAWDEPDLLQNVKRVSPWLVELVSNMPT 388
AtARF16             DSSRISWFMGTVSAVNVSDPIRWPNSPWRLLQVAWDEPDLLQNVKRVNPWLVELVSNVHP 390
OsARF18_Os06g47150  DSSRISWFMGTVSAVQVADPIRWPNSPWRLLQVSWDEPDLLQNVKRVSPWLVELVSNMPA 403
OsARF22_Os10g33940  DSSRISWFMGTISSVQVADPNRWPNSPWRLLQVTWDEPDLLQNVKCVSPWLVELVSSIPP 399
OsARF8_Os02g41800   DSSRISWFMGTVASVQVADPIRWPQSPWRLLQVTWDEPDLLQNVKRVSPWLVELVSSMPA 382
OsARF10_Os04g43910  DSSRISWFMGTVAGVQASDPVRWPQSPWRLLQVTWDEPELLQNVKRVCPWLVELVSSMPN 391
AtARF17             DSSRITWFQGIVSSTYQETG-PWRGSPWKQLQITWDEPEILQNVKRVNPWQVEIAAHATQ 384
                    *****:** * ::..        *  ***: **::****::***** * ** **:.:
```

```
AtARF10                IHLSPFSP-RKKIRIPQPFEFPFHGTKFPIFSPGFANNGGGESMCYLSN---------DN 438
AtARF16                IPLTSFSPPRKKMRLPQHPDYNNLINSIPVPS--FPSNP-LIRSSPLSS---------VL 438
OsARF18_Os06g47150     IHLAPFSPPRKKLCVPLYPELPIDG---QFPTPMFHGNPLARGVGPMCY---------FP 451
OsARF22_Os10g33940     IHLGPFSSPRKKLRVPPHPDFPFEG---HLLNPIFHGNPLGPSNSPLCC---------YP 447
OsARF8_Os02g41800      INLSSFSPPRKKPRILAYPEFPFEG--QLLN-PAFPPNPLAHGHHHYHHNHPSFFPFPDV 439
OsARF10_Os04g43910     LHLPSFSPPRKKPRNPPYAELPLEG--QIFTGPVFPPNPMAHDHHHHHG--FPFLPFPDS 447
AtARF17                LHTPFPPAKRLKYPQPGGGFLSGDDGEILYPQSGLSSAAAPDPSPSMFS----------Y 434
                       :     .. * *                      :

AtARF10                NNAPAGIQGARQ-AQQLFGSPSPSLLSDLNLSSYTGNNKLHSPAMFLSSFNPR---HHHY 494
AtARF16                DNVPVGLQGARHNAHQYYG------LSSSDLHHYYLNRPPPPPPPSSLQLSP----SLGL 488
OsARF18_Os06g47150     DGTPAGIQGARHAQFGIS-------LSDLHLNKLQSSLSPHG-LHQLDHGMQP-RIAAGL 502
OsARF22_Os10g33940     DTAPAGIQGARHAQFGLP-------LTDHQLNKLHLGLLHSGSFNRLDAITPPSRISKGF 500
OsARF8_Os02g41800      SA-PAGIQGARHAQFGPSLSDLHLTHLQS-SLMYPGLRRP--DHVGPTSIPP-PRISTDL 494
OsARF10_Os04g43910     SAQPAGIQGARHAQFASPFPEFHIGNLQPNLMLYAGIRLPPADRAAPAPRPPRIIISTDL 507
AtARF17                STFPAGMQGARQYDFGSFN------------------------PTGFIGGNPPQLFTNNF 470
                       .  *.*:****:

AtARF10                QARDSENSNNISCSLTMGNPAMVQDKKKSVGSVKTHQFVLFGQPILTEQQVMNR----KR 550
AtARF16                RNIDTKNEKGF-CFLTMGTTPCNDTKSK------KSHIVLFGKLILPEEQLSEKGSTDTA 541
OsARF18_Os06g47150     IIGHPAARDDISCLLTIGSPQNNKKSDG---KKAPAQLMLFGKPILTEQQISLGDAASVD 559
OsARF22_Os10g33940     VVSSAPAHDNISCLLSISTPQVAEKSDD---RKTTPHIMLFGKAIFTEQQITS--SGSTE 555
OsARF8_Os02g41800      TMGSS----PPARALSMGAKKPDDAKPP--------GLMLFGQRILTERQMSLSGTTSPA 542
OsARF10_Os04g43910     TIGSPGKPDDAACSPSSGGKKIDDTKPR--------GFLLFGQAILTEEQIKNGNSDGRP 559
AtARF17                LSPLPDLGKVSTEMMNFGSPPSDNLSPN-------------------------------- 498
                           .          . .     . .

AtARF10                FLEEE---AEAE------------EEKGLVARGLTWN-------------YSLQGLETGH 582
AtARF16                NIEKT---QISSGG---------SNQNGVAGREFSSSDEGS---PCSKKVHDASGLETGH 586
OsARF18_Os06g47150     VKKSS---SDGNAENTVNKSNSDVSSPRSNQNGTTDNLSCGGVPLCQDNKVLDVGLETGH 616
OsARF22_Os10g33940     TLSPG---VTGNSS-----PNGNAHKTGNASDGSGSSICIG--FSSQGHEASDLGLEAGH 605
OsARF8_Os02g41800      ATGNSSLNWNTEKG--ASEGSGSGVIQNSPTDNTS-SERLQWFREN--STVSELGLEPGQ 597
OsARF10_Os04g43910     ASPN----WDAEKAPNTSEGSDSGVTQGSPTKNTTPSWSLPYFGGNNISRASEYELNPGQ 615
AtARF17                --------------------------------------------------SNTTNLSSG- 507
                                                                      *..*

AtARF10                CKVFMESEDVGRTLDLSVIGSYQELYRKLAEMFHIEERSDLLTHVVYRDANGVIKRIGDE 642
AtARF16                CKVFMESDDVGRTLDLSVLGSYEELSRKLSDMFGIK-KSEMLSSVLYRDASGAIKYAGNE 645
OsARF18_Os06g47150     CKVFMQSEDVGRTLDLSVVGSYEELYRRLADMFGIE-KAELMSHVFYRDAAGALKHTGDE 675
OsARF22_Os10g33940     CKVFMESEDVGRTIDLSVFGSYEELYGRLADMFGIE-KEEIINHLHFRDAAGVVKHPGEV 664
OsARF8_Os02g41800      CKVFIESDTVGRNLDLSSLASFEQLYGRLSEMFCID-SAELRSRVLYRGATGEVRHAGDE 656
OsARF10_Os04g43910     CKVFVESETVGRSLDLSALSSFEELYACLSDMFSIG-SDELRSHLVYRSPAGEVKHAGDE 674
AtARF17                ------NDLVGNRGPLSKKVNSIQLFGKIITVEEHSESGPAESGLCEEDGSKESSDNETQ 561
                             .: **.   **   .  :*   :  :          . :  ..

AtARF10                PFSDFMKATKRLTIKMDIGGDNVRKTWITGIRTGENGIDASTKTGPLSIFA 693
AtARF16                PFSEFLKTARRLTILTEQGSESVVV-------------------------- 670
OsARF18_Os06g47150     PFSEFTKTARRLNILTDTSGDNLAR-------------------------- 700
OsARF22_Os10g33940     PFSDFMKAARRLTIIAGDR-ERIERPLIECLVEQA---------------- 698
OsARF8_Os02g41800      PFSEFIKLARRLTILTDAGSDNLGS-------------------------- 681
OsARF10_Os04g43910     PFCAFVKSARKLRILTDAGSDNLGD-------------------------- 699
AtARF17                LSLSHAPPSVPKHSNSNAGSSSQG--------------------------- 585
                           .   :           .
```

Figure S2. Alignment of proteins encoded by miR160 target genes in *Arabidopsis* and by *Os*miR160 potential target genes in rice.

ClustalW2 was used for sequence alignment. Sequences were retrieved from the NCBI database.

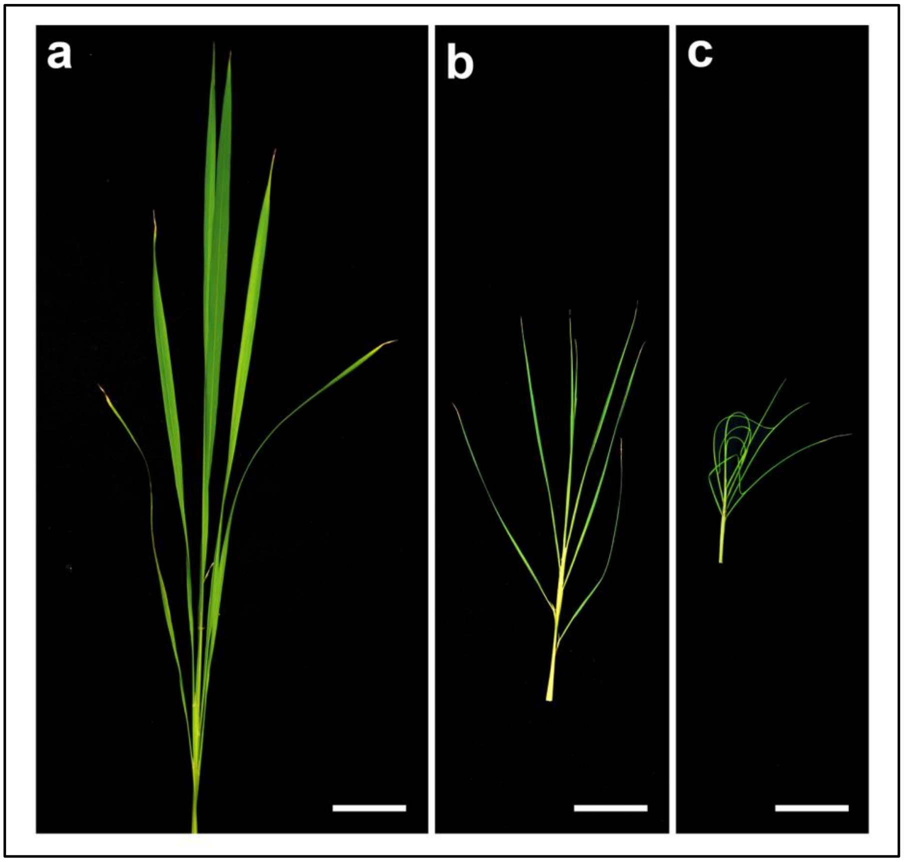

Figure S3. Abnormal growth of *mOsARF18* plants.

(**a-c**) Main shoots of wild-type (a), *mOsARF18-3* (b), and *mOsARF18-5* (c). Bars: (a-c) 5 cm.

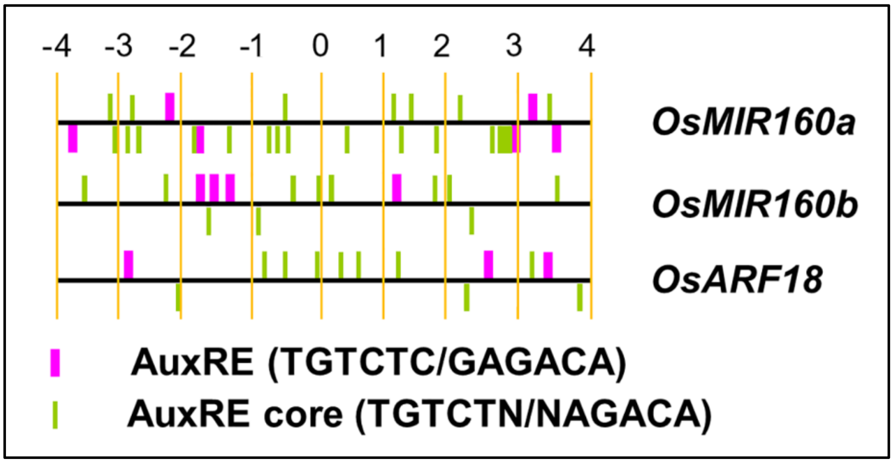

Figure S4. A diagram showing predicated *cis* elements AuxREs (auxin response elements) and AuxRE cores in *OsMIR160a*, *OsMIR160b,* and *OsARF18* genes. Number 0 indicates

transcription start sites. Other numbers indicate kilobase(s) of upstream and downstream nucleotides.

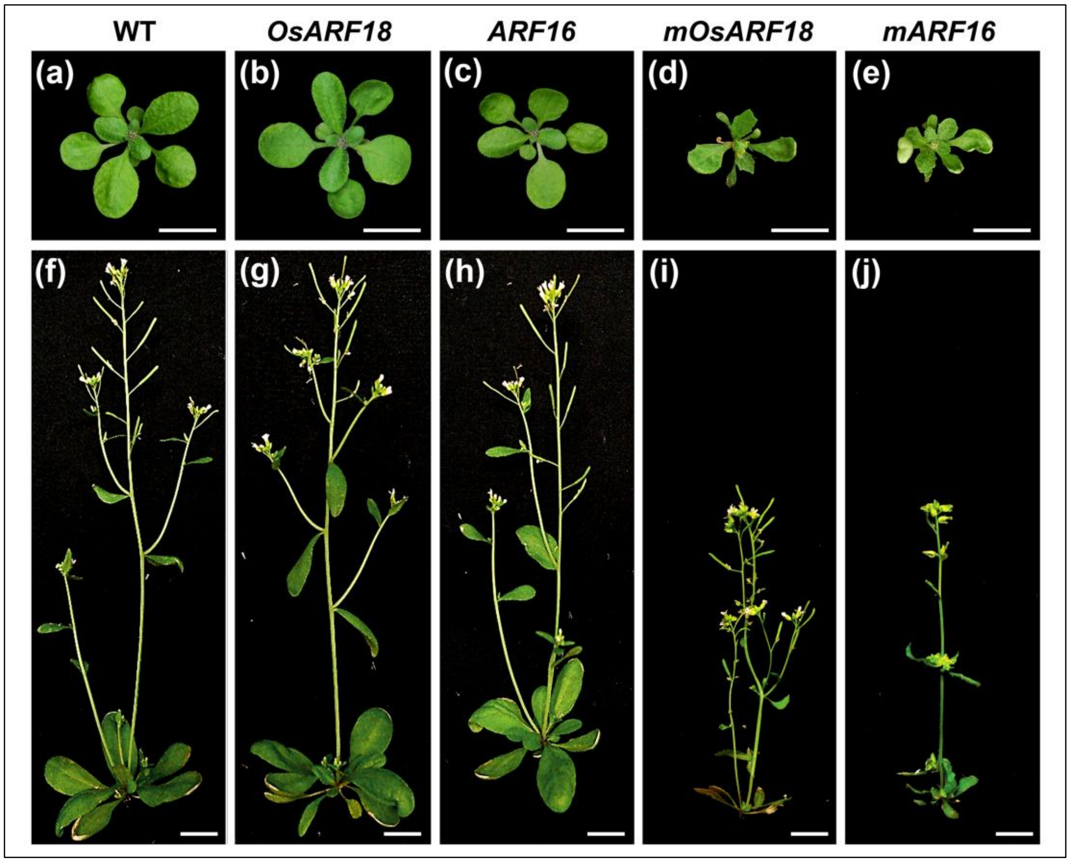

Figure S5. Ectopic expression of *mOsARF18* and *mARF16* in *Arabidopsis* caused abnormal growth.

OsARF18, ARF16, mOsARF18, and *mARF16* were expressed in *Arabidopsis* under control of the *Ubi* promoter. (a-e) Three-week old wild-type (a), *OsARF18* (b), *ARF16* (c), *mOsARF18* (d), and *mARF16* (e) plants. Bars: (a-e) 1 cm. (f-j) Five-week old wild-type (f), *OsARF18* (g), *ARF16* (h), *mOsARF18* (i), and *mARF16* (j) adult plants. Bars: (f-j) 1 cm.

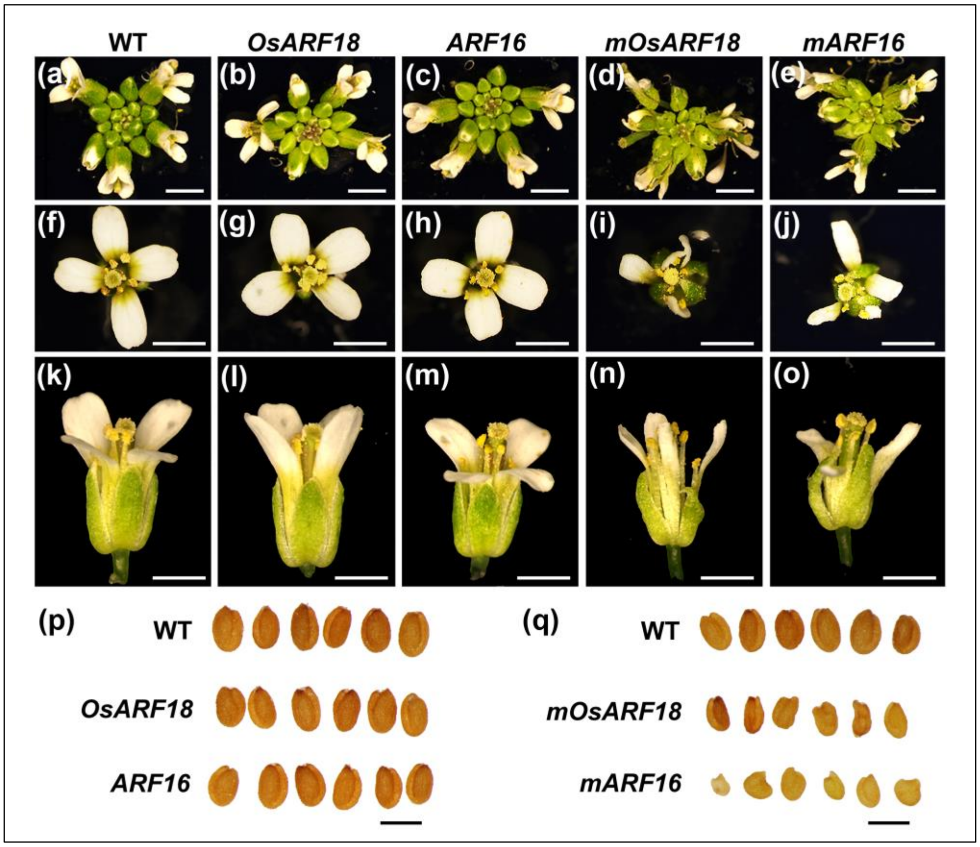

Figure S6. Ectopic expression of *mOsARF18* and *mARF16* in *Arabidopsis* resulted in abnormal reproductive development.

OsARF18, ARF16, *mOsARF18,* and *mARF16* were expressed in *Arabidopsis* under control of the *Ubi* promoter. (a-e) Five-week old wild-type (a), *OsARF18* (b), *ARF16* (c), *mOsARF18* (d), and *mARF16* (e) inflorescences. Bars: (a-e) 2 mm. (f-o) Top and side view of wild-type (f, k), *OsARF18* (g, l), *ARF16* (h, m), *mOsARF18* (i, n), and *mARF16* (j, o) flowers, respectively. Bars: (f-o) 1 mm. (p, q) Mature seeds of wild-type, *OsARF18* and *ARF16* (p), as well as of wild-type, *mOsARF18* and *mARF16* (q). Bars: (p, q) 0.5 mm.

Table S1 Primers used in this study.

Primer	Primer name	Purpose	Sequence (5' to 3')
ZP1804	*OsARF18* cDNA f	*mOsARF18*	CACCATGATAACTTTCGTGGATTCCGC
ZP1805	Overlapping PCR	*mOsARF18*	ATGGCACTCCTGCAGGAATTCAAGGTGCAC GGCATGCTCAATTTGGTATATCTTTA
ZP1806	Overlapping PCR	*mOsARF18*	ACCAAATTGAGCATGCCGTGCACCTTGAATT CCTGCAGGAGTGCCATCC
ZP1807	*OsARF18* cDNA r	*mOsARF18*	CCTCGCTAAGTTATCGCCGC
ZP1246	*ARF16* cDNA f	*ARF16 or mARF16*	ATGATAAATGTGATGAAT
ZP1297	Overlapping PCR f	*mARF16*	CGTGGGTTTGCAAGGGGCAAGACATAAT GCTCATCAGTACTACGG
ZP1298	Overlapping PCR r	*mARF16*	ATTATGTCTTGCCCCTTGCAAACCCACGG GAACATTGT
ZP1247	*ARF16* cDNA r	*ARF16 or mARF16*	TTATACTACAACGCTCTCACTTCCT
ZP2256	GeneRacer™ RNA Oligo Sequence	5' RACE	CGACUGGAGCACGAGGACACUGACAUGG ACUGAAGGAGUAGAAA
ZP2257	GeneRacer™ 5′ Primer	5' RACE	CGACTGGAGCACGAGGACACTGA
ZP2258	GeneRacer™ 5′ Nested Primer	5' RACE	GGACACTGACATGGACTGAAGGAGTA
ZP2259	*OsARF18* gene specific reverse primer	5' RACE	CATCTCCAGTGTGCTTGAGTGCCCC
ZP2033	*OsMir160a* f	qRT-PCR	GCCTGCATATGGTTGCTTGAC
ZP2034	*OsMir160a* r	qRT-PCR	CACATCCTTCTTGCACCTGC
ZP2035	*OsMir160b* f	qRT-PCR	CCGTTTGGATATGTGTTTGTCTGC
ZP2036	*OsMir160b* r	qRT-PCR	GCTGCTCTCCATGCTGATC
ZP2037	*OsMir160c* f	qRT-PCR	CTAGAGCAACAAACTTCTGCGAG
ZP2038	*OsMir160c* r	qRT-PCR	GGAGAAGGTTGGAAAATAGGAGGG
ZP2039	*OsMir160d* f	qRT-PCR	CTCAGTTGAAACAACTGCCTTCTC
ZP2040	*OsMir160d* r	qRT-PCR	GCACACACAATGAGTCAATGAGAG
ZP2041	*OsMir160e* f	qRT-PCR	GCATGGCCCTCTTCCCTTC
ZP2042	*OsMir160e* r	qRT-PCR	GCACTGTCATGTTCATATCCACTG
ZP2043	*OsMir160f* f	qRT-PCR	GCAAGCACGGCTTCCTAG
ZP2044	*OsMir160f* r	qRT-PCR	GCGCTGTCCTTTTAGTCTTCC
ZP2058	*OsARF8* UCf	qRT-PCR	GCCACCACCATTACCATCAC
ZP2059	*OsARF8* UCr	qRT-PCR	GGTGGGTAAGGTGGAGATCTG

ZP2050	*OsARF10* UCf	qRT-PCR	CCACCACCACCACCATG
ZP2051	*OsARF10* UCr	qRT-PCR	CAGGTCGGTGCTGATGATG
ZP2046	*OsARF18* UCf	qRT-PCR	CCACCTTGCTCCATTCTCG
ZP2047	*OsARF18* UCr	qRT-PCR	GCAGTTTGTTAAGGTGGAGATCTG
ZP2054	*OsARF22* UCf	qRT-PCR	CCACATCCTGACTTCCCATTTG
ZP2055	*OsARF22* UCr	qRT-PCR	GTAACAGACCAAGGTGCAGC
ZP2064	*OsPIN1b* f	qRT-PCR	CACCAACAACCCGTACACGATG
ZP2065	*OsPIN1b* r	qRT-PCR	CACTGCAGCACGACGATCTG
ZP2066	*OsLAX2* f	qRT-PCR	GATCTACCTGATGGCGACGC
ZP2067	*OsLAX2* r	qRT-PCR	CTTGAAGAGGCTGCGGCAG
ZP2068	*OsYUCCA2* f	qRT-PCR	GTCCAAAGGGAGGAGTCCAG
ZP2069	*OsYUCCA2* r	qRT-PCR	CATGATGTTTACACCCGGCCT
ZP2070	*OsARF2* f	qRT-PCR	GCACTCTCCAGTCAGTGGTG
ZP2071	*OsARF2* r	qRT-PCR	GATAACTGGCATTGCCACAATCCAC
ZP2060	*OsGH3-1* f	qRT-PCR	CAGCCCGCACAAGAAGTGATC
ZP2061	*OsGH3-1* r	qRT-PCR	CTACCACTGGACAGCATGATCTAAG
ZP1950	*OsUBQ5* f	qRT-PCR	GCACAAGCACAAGAAGGTGA
ZP1951	*OsUBQ5* r	qRT-PCR	TCGATTTCCTCCTCCTTCCT
